# Use of Parathyroid Function Index and Wisconsin Index to Differentiate Primary Hyperparathyroidism From Secondary Hyperparathyroidism: A Case-Control Study

**DOI:** 10.7759/cureus.23043

**Published:** 2022-03-10

**Authors:** Murat Bulut Özkan, Veysel Barış Turhan

**Affiliations:** 1 General Surgery, T.R. Ministry of Health Hitit University Çorum Erol Olçok Training and Research Hospital, Çorum, TUR

**Keywords:** pfindex, vitamin-d deficiency, secondary hyperparatiodism, parathyroid function test, primary hyperparathyroidism

## Abstract

Introduction: Patients with primary hyperparathyroidism (PHPT) can be asymptomatic or have a normal calcium level (NHPT). Patients with 25(OH) vitamin D insufficiency, on the other hand, may present with a similar presentation. In regions where 25(OH) vitamin D deficiency is common, patients are usually diagnosed with secondary hyperparathyroidism (SHPT). Therefore, it is necessary to separate PHPT and NHPT from SHPT. Parathormone and calcium values are used for differentiation in the clinic. The predictive value of the newly developed parathyroid function test (PFindex), which previously had a high diagnostic value, was evaluated in this patient population in our investigation.

Methods: The study comprised 163 PHPT and NHPT patients with pathological confirmation and 56 SHPT patients. The PHPT, NHPT, and SHPT properties were defined using PFindex. The diagnostic power of PFindex was investigated using a receiver operating characteristic (ROC) curve of the results assessed in three groups.

Results: The PHPT group had the highest PFindex (1365.4±784.6) compared to the other two groups (NHPT: 723.5±509.4; SHPT:227.2±49.9, all p < 0.001). A PFindex threshold of 327.8 yielded 91.9% and 90.9% sensitivity and specificity rates for distinguishing PHPT and NHPT from SHPT, respectively.

Conclusion: PFindex gave the outstanding diagnostic capacity to distinguish PHPT from SHPT due to our research. This straightforward tool can assist in making quick decisions about vitamin D therapy or surgery for PHPT.

## Introduction

Parathyroid hormone (PTH) is an 84-amino-acid polypeptide hormone produced by the pituitary gland. It is released by the parathyroid glands and is one of the most essential hormones for maintaining blood calcium (Ca) and phosphate (PO4) balance. It causes 25(OH)D to be hydroxylated to 1,25(OH)2D, resulting in intestinal calcium absorption and supporting calcium and phosphate release from the bone. As a result, the blood calcium level rises [1,2].

Primary hyperparathyroidism (PHPT) is a condition that affects calcium metabolism due to parathyroid hormone (PTH) hypersecretion in one or more of the four parathyroid glands. The abnormal elevation of PTH leads to increased serum calcium [3,4]. In fact, PHPT is considered the most common cause of hypercalcemia in ambulatory patients [5].

The biochemical, skeletal, and renal signs of classic PHPT can all be used to diagnose. However, in recent decades, an increase in the detection frequency of asymptomatic PHPT (including normocalcemic PHPT, NHPT) has been attributed to more regular serum calcium monitoring and the inadvertent identification of parathyroid nodules on thyroid ultrasonography [6,7]. As a result, a proper clinical diagnosis of this condition is critical.

Chronic renal insufficiency and vitamin D deficiency are the most common causes of secondary hyperparathyroidism (SHPT) [8]. Native vitamin D [25(OH)D)] has a hormonal system that regulates calcium homeostasis and bone metabolism. Not just in certain kinds of patients but also in the general population, 25(OH)D insufficiency is fairly frequent [9]. In Europe, vitamin D insufficiency is fairly frequent, too [10]. McKenna [11] observed that during the winter, 40% of native young, healthy individuals in Central and Southern Europe suffered from vitamin D insufficiency, which was frequently associated with increased PTH levels. Furthermore, 14% of healthy adult volunteers had 25(OH)D insufficiency and increased PTH levels in a major French research [12].

Based on test data, it might be challenging to differentiate SHPT caused by vitamin D insufficiency from PHPT. Both SHPT and PHPT patients have high PTH levels. Furthermore, most individuals have normal blood calcium levels and low 25(OH)D levels [13,14].

In a recent paper, Guo et al. [15] created a handy tool called the parathyroid function index (PFindex) to distinguish PHPT from SHPT. PHPT is related to a greater blood calcium concentration but a lower phosphate concentration, whereas SHPT has lower serum calcium and phosphate values. They developed PFindex based on this data to highlight the biochemical differences between these illnesses. This equation divides the blood phosphate concentration by serum calcium concentration and then multiplies the serum PTH.

Our study aims to re-examine the power of this diagnostic tool, which has been published only once in the literature and has a high predictive value in a population where vitamin D deficiency is common.

## Materials and methods

Study population

This retrospective case-control research includes 219 patients. Between January 10, 2015, and October 1, 2021, 163 PHPT and NHPT patients were identified pathologically and surgically, and 56 SHPT patients were reviewed after receiving approval number 2021-81 dated 09/11/2021 from the T.R. Hitit University Non-Interventional Research Ethics Committee and was conducted by the Helsinki Declaration Principles. Three groups of biochemical parameters were compared using data from computer records, surgical notes, and patient files.

Preoperative parameters were recorded at the time of PHPT diagnosis and before initiating any special treatment to be included in the PHPT group: age, gender, serum Ca, P, creatinine, PTH, and 25(OH)D. Another subgroup was created by choosing NHPT patients from the PHPT cohort (Table 1).

**Table 1:** Characteristics of subjects in Primary HPT, Normocalcemic HPT, Secondary HPT groups *Kruskal-Wallis test; M: Male; F: Female; WIN: Wisconsin Index; PTH: Parathormone; HPT: Hyperparathyroidism

	Primary HPT(n=121)	Normocalcemic HPT(n=52)	Secondary HPT(n=56)	p value
Age, years, mean±SD	55.8±12.8	56.1±11.8	55±13.6	0.932*
Gender(M/F)	31/130	20/53	38/50	0.352*
Serum corrected calcium (8.6-10.2 mg/dL), mean±SD	11.1±2.4	9.8±1.3	9.3±0.3	<0.001*
Serum phosphate(2.5-4.5 mg/dL), mean±SD	2.1±0.5	2.6±0.6	3.3±0.4	<0.001*
PTH(15-65 pg/mL), mean±SD	242±120.3	167.2±90.5	97.6±15.2	<0.001*
25(OH)D (ng/mL), mean±SD	13.5±3.3	15.5±5.1	21.8±2.8	<0.001*
Calcium*PTH(WIN), mean±SD	2721.8±1535.1	1774.2±1117.4	912.4±142.3	<0.001*
Calcium/Phosphate, mean±SD	5.7±1.8	4.1±1.2	2.8±0.3	<0.001*
PFindex, mean±SD	1365.4±784.6	723.5±509.4	227.2±49.9	<0.001*

Fifty-six age-matched SHPT cases were selected from hospital computer records. Subjects with serum PTH concentration > 65 ng/mL and 25(OH)D concentration < 30 ng/mL were classified into the SHPT group. Corrected calcium was calculated as the measured calcium + [(4 − measured albumin) x0.8] [16]. The PHPT, NHPT, and SHPT properties were defined using the PFindex and Wisconsin Index (WIN). WIN was defined as the multiplication of preoperative serum calcium by preoperative parathyroid hormone. The PFindex was defined as multiplying the preoperative serum calcium with the preoperative parathyroid hormone and dividing the value by phosphate.

Statistical analysis

The SPSS program for Windows version 22.0 was used to assess the data analysis (IBM Corp., Armonk, NY, USA). The mean + standard deviations represent continuous variables having a normally distributed distribution. The Kolmogorov-Smirnov test was used to demonstrate that the parameters followed a normal distribution. The diagnostic capacity of PFindex was evaluated using a receiver operating characteristic (ROC) curve analysis, and ROC curves were generated to analyze the balance between sensitivity and specificity. P-value 0.05 was used to determine statistical significance.

## Results

**Figure 1:**
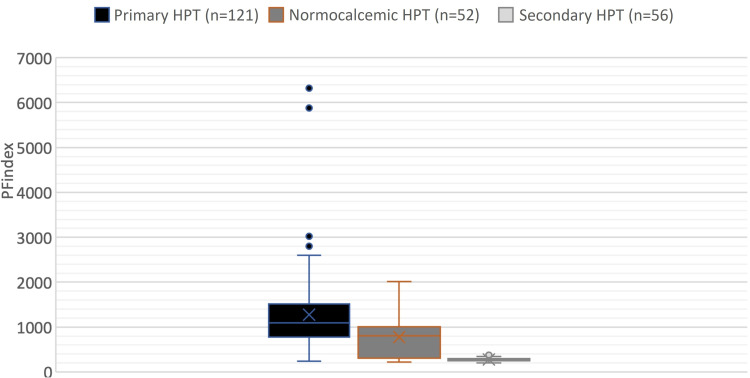
Pfindex value in 3 groups

A total of 219 patients took part in the research. There were 121 PHPTs, 52 NHPTs, and 56 SHPTs among these individuals. In terms of age and gender, there was no statistical difference in the distribution of the groups (0.932, 0.352, respectively). The PHPT group had the highest adjusted serum corrected calcium, PTH, and PFindex values (all P 0.001). The SHPT has a greater 25(OH)D level than the PHPT and NPHPT (Table 1, Figure 1).

**Figure 2:**
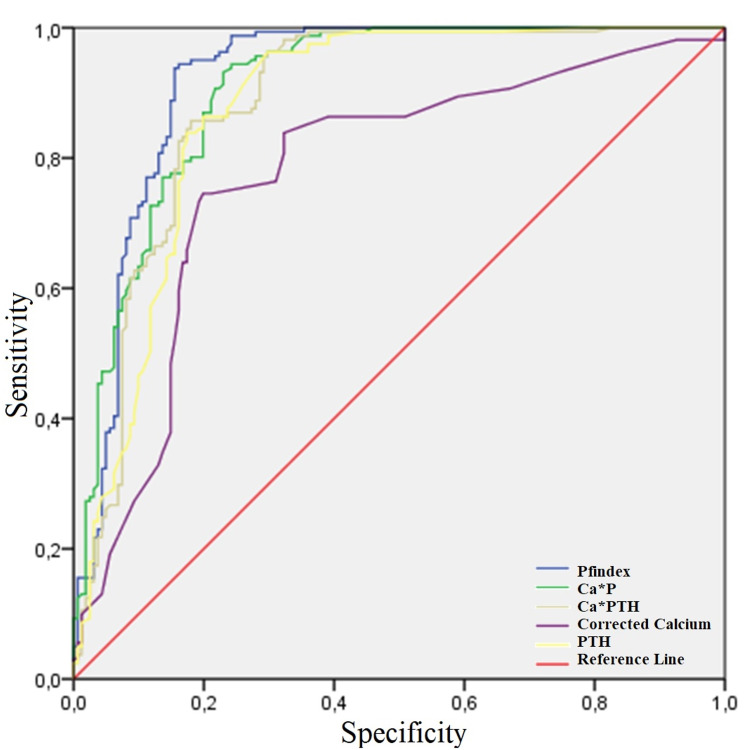
ROC Curve for Indices and Laboratory Parameters Ca: Calcium; P: Phosphate; PTH: Parathyroid hormone; ROC: Receiver operating characteristic; Wisconsin(WIN) index: Ca*PTH

The Area Under the ROC Curve (AUC) for Pfindex in the ROC analysis for the diagnosis of PHPT was 0.951. Accordingly, it provided 91.9% sensitivity and 90.9% specificity above the cut-off value of 327.8 (Figure 2). The AUC, sensitivity, and specificity rates of the PFindex curve were greater than those of the serum calcium, phosphate, PTH, and CalciumPTH curves (Table 2, Table 3).

**Table 2:** Sensitivity, Specificity, and Area Under the Curve of serum corrected calcium, PTH, Calcium×PTH, and Pfindex in diagnosis of PHPT WIN: Wisconsin Index; PTH: parathormone, AUC: Area Under the Curve

	Cut-off value	Sensitivity (%)	Specificity (%)	AUC
Pfindex	327.8	91.9	90.9	0.951
Calcium/Phosphate	3.3	89.7	88.6	0.944
Calcium*PTH(WIN)	1040.9	89.7	88.6	0.942
PTH (pg/mL)	109.5	87.6	87.5	0.938
Corrected calcium (mg/dL)	9.5	78.6	79.5	0.835

**Table 3:** Sensitivity, Specificity, and Area Under the Curve of serum corrected calcium, PTH, Calcium×PTH, and Pfindex in diagnosis of NHPT WIN: Wisconsin Index; PTH: parathormone; AUC: Area Under the Curve

	Cut-off value	Sensitivity (%)	Specificity (%)	AUC
Pfindex	327.8	91.2	90.9	0.932
Calcium/Phosphate	3.3	71.2	85.2	0.799
Calcium*PTH(WIN)	1040.9	80.1	89.2	0.834
PTH (pg/mL)	109.5	74.4	89	0.826
Corrected calcium (mg/dL)	9.5	58.6	61.5	0.319

## Discussion

The predictive value of Pfindex, which was examined for the first time by Guo et al. [15], was re-examined. According to our findings, this test is useful for differentiating PHPT patients from SHPT patients. Our results also revealed that PFindex outperformed the previously developed Wisconsin Index in terms of AUC, specificity, and sensitivity, and the Ca/P ratio outperformed both.

The Wisconsin Index was defined as the multiplication of preoperative serum calcium by preoperative parathyroid hormone (PTH) by Mazeh et al. [17] in 2013. However, in this study, the Wisconsin Index was designed to decide whether to expect an intraoperative PTH value during parathyroidectomy and whether a more comprehensive exploration would be needed in parathyroidectomy, rather than the differential diagnosis of PHPT. In addition, two studies published in 2021 evaluated the usefulness of WIN in predicting monoglandular and multiglandular disease in PHPT, and it was shown that it is not as helpful as intraoperative PTH in predicting the multiglandular disease [18,19]. In this study, WIN was used for the first time for differential diagnosis. As a result of our research, sensitivity was found to be 89.7% and specificity 88.6% for WIN in distinguishing PHPT patients and a cut-off value of 1040.9 (AUC: 0.942). In addition, the sensitivity of 80.1% and specificity of 89.2% and a cut-off value of 1040.9 (AUC: 0.834) were found for WIN in differentiating NPHPT patients.

Madeo et al. examined the Ca/P value in three different studies. They concluded that in the first of these studies, Ca/P above 3.56 is a reasonably accurate tool for identifying PHPT and HypoP in patients not associated with PHPT [20]. Another study found that a Ca/P ratio above 2.55 was a PHPT and a value below 1.78 was a very accurate index to define hypoparathyroidism. Sensitivity was 85.7% and specificity 85.3% in differentiating PHPT patients [21]. As a result of other studies, Ca/P correctly identified 441 of the 590 (74.7%) PHPT records. With a cut-off of 2.64 for the Ca/P ratio, the diagnostic power of the Ca/P ratio showed 80% sensitivity and 88% specificity [22]. In our study, sensitivity and specificity values were higher for the 3.3 value (89.7%, 88.6%, respectively).

PFindex was first described by Guo et al. [15]. As a result of their study, the PF index was a combination of the previously discussed WIN and Ca/P values. As a result of their research, the diagnostic power of PFindex was stronger than the Ca/P Ratio and the Wisconsin Index. As a result of their studies, the sensitivity was 96.9%, and the specificity was 97.6% for the value with PFindex > 34 in PHPT discrimination (Youden index: 0.945). Similarly, in our study, the superiority of the PFindex in differential diagnosis was indisputable. For a cut-off value of 327.8, sensitivity was 91.9% and specificity was 90.9%. The reason for the difference in the cut-off value is due to the difference in the unit part of the values.

WIN had a lower diagnostic value in our investigation for the diagnosis of PHPT and NHPT and the differential diagnosis of SHPT. This is because it ignores the influence of phosphate hemostasis. Also, compared to WIN and PFindex, Ca/P ratios exhibited a lower differential diagnostic value. Although a high PTH level with a high or normal calcium level is commonly used to diagnose PHPT, the Ca/P ratio ignores the PTH level.

The symptoms of PHPT are dependent on a high calcium level. Patients in studies are typically symptomatic, but there are also asymptomatic patients [6,7,23]. Vitamin D deficiency has been found in PHPT patients in previous studies, and vitamin D deficiency causes low serum calcium levels [24,25]. Patients may be asymptomatic as a result of this. It takes a long time to treat patients with elevated PTH due to vitamin D deficiency [26]. Patients with a cut-off value higher than those obtained in our study can be diagnosed with PHPT without waiting for the waiting period to expire. Furthermore, in patients with a vitamin D deficiency, the disease can be treated with vitamin D supplementation without the need for further testing. As a result, easily calculated values like PFindex and WIN can save time and money while aiding in the differential diagnosis of disease.

Our study has limitations due to its retrospective nature. In addition, since there is a general vitamin D deficiency in Turkish society, this may be reflected in the data [27]. The low number of patients is another limitation. but we had twice the patient population compared to the previous study. We did not use healthy individuals as a control group. It was difficult to reach these data in our study.

## Conclusions

The results obtained from the blood values obtained routinely in this patient population are suitable for use in centers that do not have the opportunity for further examination and are financially challenged, considering the high cost of further examinations. In addition, we believe that these indexes may be the gold standard as a result of prospective randomized studies.
